# Pregnancy rate and birth outcomes among women receiving antiretroviral therapy in Burkina Faso: a retrospective cohort study

**DOI:** 10.11604/pamj.2016.23.105.7372

**Published:** 2016-03-16

**Authors:** Armel Poda, Arsène Hema, Aina Konaté, Firmin Kaboré, Jacques Zoungrana, Euloges Kamboulé, Ibrahim Soré, Guillaume Bado, Abdoul-Salam Ouédraogo, Macaire Ouédraogo, Nicolas Meda, Adrien Bruno Sawadogo

**Affiliations:** 1 Hôpital de Jour, Service des Maladies Infectieuses, CHU Souro Sanou, Bobo Dioulasso, Burkina Faso; 2 Institut Supérieur des Sciences de la Santé (INSSA), Université Polytechnique de Bobo Dioulasso (UPB), Bobo Dioulasso, Burkina Faso; 3 Service de Bactériologie Virologie, CHU Souro Sanou, Bobo Dioulasso, Burkina Faso; 4 Service de Médecine Interne, CHU Souro Sanou, Bobo Dioulasso, Burkina Faso; 5 Unité de Formation et de Recherche en Sciences de la Santé, Université de Ouagadougou, Ouagadougou, Burkina Faso; 6 Centre Muraz, Bobo Dioulasso, Burkina Faso

**Keywords:** ART, fertility, HIV, pregnancy incidence, Burkina Faso

## Abstract

**Introduction:**

In Sub-Saharan Africa, few studies reported pregnancy incidence and outcomes in women taking antiretroviral therapy (ART). This survey aims to estimate the incidence and outcomes of pregnancy in a cohort of HIV positive women initiating ART in Bobo-Dioulasso, Burkina Faso.

**Methods:**

We carried out a retrospective cohort study. We selected women in childbearing age initiating ART and followed up in Bobo-Dioulasso teaching hospital between January 2005 and June 2011. The incidence of pregnancies during follow-up was calculated. Childbirth was defined by the expulsion of a fetus after 22 weeks of amenorrhea. Before this term, it is an abortion. Childbirth is said premature if it occurs before 37 weeks of gestation, to term if it occurs between the 38th and the 42nd week. The annual age-standardized fertility rates were calculated using the baseline population from the 2010 demographic and health survey (DHS) in Burkina Faso.

**Results:**

A total of 1,763 women of childbearing age under ART were included in the study. They ranged between 18 and 48 years old with a median of 35 years old. A total of 222 pregnancies were observed during 4639 women-years of follow-up, corresponding to an incidence density of 5 pregnancies for 100 women-years (95% CI: 4.2-5.5). Among the 222 pregnancies recorded, 9(4.0%) ended with abortion, 205(92.4%) with childbirth (including 15 premature childbirths); the outcome of 8(3.6%) pregnancies were unknown abortion. Live birth and stillborn rates were 94.0% (193/205) and 6.0% respectively. The standard fertility rate in our cohort was 45 live births for 1,000 women-years. The general decrease in fertility rates was 66.0% among women infected with HIV compared to the overall population

**Conclusion:**

This study shows a low pregnancy incidence among women initiating ART as compared to their peers from the general population. Pregnancies that occurred during ART generally end with live births. Care packages for HIV infected women of childbearing age must include reproductive health services to better address this issue.

## Introduction

Sub-Saharan Africa is the most HIV affected area, with women as the most vulnerable population. Indeed, 58% of people living with HIV/AIDS (PLWHA) in Sub-Saharan Africa was women of childbearing age [1]. Improving life expectancy and quality for PLWHA on antiretroviral therapy (ART) increases their desire for bearing children [2–7] even though HIV infection is associated with a 25 to 40% decrease in fertility among women of childbearing age [8–10]. Pregnancy is not likely to influence the progression of the infection [11]. Additionally, as long as the mother is in good health, HIV does not impact the course of the pregnancy [12]. The risks of complications are higher for the mother and her fetus when mother develops an HIV-related opportunistic disease and when the CD4 cells count is below 200/µl [13]. Miscarriages, abortions, ectopic pregnancies and stillborns delivery seem to be as recurrent among HIV positive women (some of whom were under ART) as among non infected women [8, 14–16]. According to the UNAIDS Report on Global AIDS Epidemic, 60% of HIV-infected people in Burkina Faso are women of childbearing age [17]. Besides, the desire for bearing children is quite high among women in general in Burkina Faso including HIV infected women [18]. However, there are little data on the frequency of pregnancies among HIV-infected women under antiretroviral treatment. We therefore deemed it necessary to study the incidence and outcome of pregnancies in Bobo-Dioulasso Day Hospital cohort. Knowing the status of women under ART who get pregnant will help provide appropriate care to countries with limited resources.

## Methods

***Framework and period of the study:*** the study was conducted from January 1st, 2005 to June 30, 2011 at the Souro Sanou Teaching Hospital, Bobo-Dioulasso, a public referral hospital in Burkina Faso offering outpatient care services to people living with HIV [19].

***Type of study:*** this is a retrospective cohort study.

***Targeted population:*** patients targeted in the study include non pregnant women of childbearing age (18- 49 years) under antiretroviral treatment between January 2005 and December 2010. They were followed up until June 30, 2011 (reference date).

***Data collection:*** data for the follow up of people living with HIV were collected at Bobo-Dioulasso day hospital using the ESOPE software that was regularly filled in by doctors during medical visits. The socio-demographic, biological and therapeutic data of the study were taken from ESOPE database. For the diagnosis of pregnancies, immunologic tests (BBtestR) were conducted at the beginning of the antiretroviral treatment and at the occurrence of amenorrhea during the follow up. Positive pregnancy tests were confirmed by pelvic scan. Pregnancy development and outcome-related data were collected from patients’ antenatal care records. Childbirth was defined by the expulsion of a fetus after 22 weeks of amenorrhea. Before this term, it is an abortion. Childbirth is said premature if it occurs before 37 weeks of gestation, to term if it occurs between the 38th and the 42nd week.

***Data analysis:*** the data collected were analyzed using STATA version 12. A description of the socio-demographic, biological and clinical characteristics of the survey population was made. Common statistics including the median and inter quartile range (IQR) and percentages (%) were used to describe the survey population. The exact Fischer test was used to compare proportions, while Student t test served to compare continuous variables. The pregnancy incidence was estimated from the number of pregnancies based on the sum of observation periods for each patient. Only the first pregnancies under ART were considered in calculating the incidence. The pregnancy occurrence risk according to the ART time was estimated using the Kaplan Meier method. The outcome of pregnancies was described. The fertility rate in terms of number of live births for 1,000 women was estimated in the survey population. The annual age-standardized rates (indirect standardization) were calculated using the baseline population from the 2010 demographic and health survey (DHS) in Burkina Faso [10]. The agreed significance threshold was 5% for all analyses.

***Ethical considerations:*** this observational study was conducted as part of the routine care services and therefore presented no risk to patients. For confidentiality purposes, the data collected were made anonymous.

## Results

***Socio-demographic characteristics of the survey population:*** On December 31st, 2010 a total number of 2,717 patients including 74.0% women were under antiretroviral treatment. Between January 2005 and December 2010, a total of 1,763 women of childbearing age under ART were included in the cohort survey. They were aged between 18 and 48 years with a median age of 35 years (IQR: 30-40). Almost half of the patients (49.0%) were aged between 30 and 39. Table 1 summarizes the socio-demographic characteristics of the patients. They were mainly uneducated (42.5%; n=749), unemployed (75.7%; n=1334), almost 44.6% (n=787) living as a couple. The average number of children per woman was 2 (IQR: 2-3) with 0 and 9 children as the extremes. More than half of the women (52%; n=919) had at least two children.

**Table 1:** Women characteristics by age at the beginning of ART in Bobo Dioulasso Day Care Unit, Souro Sanou Teaching Hospital, Burkina Faso, 2005-2011

Characteristics	Age (years)							Total (n=1763)
	15 – 19 (n=8)	20 - 24 (n=98)	25 - 29 (n=300)	30 - 34 (n=416)	35 - 39 (n=448)	40 - 44 (n=299)	45- 49 (n=194)	
**Profession (%)**								
**Unemployed**	4(50.0)	74(75.5)	225(75.0)	315(75.7)	333(74.3)	233(77.9)	150(77.3)	1334(75.7)
**Trader**	0	8(8.2)	25(8.3)	42(9.9)	42(9.4)	25(8.4)	20(10.3)	161(9.1)
**Civil servant**	0	2(2.0)	18(6.0)	30(7.2)	42(9.4)	26(8.7)	11(5.7)	129(7.3)
**Other**	4(50.0)	14(14.3)	32(10.7)	30(7.2)	31(6.9)	15(5.0)	13(6.7)	139(7.9)
**Level of education (%)**								
**None**	3(37.5)	42(42.9)	105(35.0)	158(38.0)	191(42.6)	146(48.8)	104(53.6)	749(42.5)
**Primary school**	2(25.0)	38(38.8)	102(34.0)	134(31.7)	112(25.0)	88(29.4)	63(32.5)	537(30.5)
**Secondary school or higher**	3(37.5)	16(16.3)	90(30.0)	124(29.8)	139(31.0)	63(21.1)	23(11.9)	458(26.0)
**No information**	0	2(2.0)	3(1.0)	2(0.5)	6(1.4)	2(0.7)	4(2.0)	19(1.0)
**Marital status (%)**								
**Living in couple**	4(50.0)	37(37.8)	134(44.7)	211(50.7)	207(46.2)	123(41.1)	71(36.6)	787(44.6)
**Single**	3(37.5)	37(37.8)	107(35.7)	97(23.3)	82(18.3)	37(12.4)	14(7.2)	377(21.4)
**Widow**	1(12.5)	20(20.4)	37(12.3)	79(19.0)	127(28.4)	108(36.1)	86(44.3)	458(26.0)
**Divorced**	0	4(4.0)	22(7.3)	29(7.0)	32(7.1)	31(10.4)	23(11.9)	141(8.0)
**Number of live children (%)**								
**0 – 1**	8(100)	72(73.5)	210(70.0)	213(51.2)	165(36.8)	84(28.1)	30(15.5)	782(44.4)
**2 – 4**	0	19(19.4)	67(22.3)	184(44.2)	235(52.5)	158(52.8)	96(48.0)	756(42.9)
**>4**	0	0	2(0.7)	8(1.9)	31(6.9)	54(18.1)	68(35.0)	163(9.2)
**No information**	0	7(7.1)	21(7.0)	11(2.6)	17(3.8)	3(1.0)	3(1.5)	62 (3.5)
**Type of HIV (%)**								
**HIV-1**	8(100)	94(95.9)	289(96.3)	398(95.7)	429(95.8)	273(91.3)	170(87.6)	1661(94.2)
**HIV-2**	0	0	2(0.7)	4(1.0)	7(1.5)	6(2.0)	13(6.7)	32(1.8)
**HIV-1+2**	0	4(4.1)	9(3.0)	14(3.3)	12(2.7)	20(6.7)	11(5.7)	70(4.0)
**Initial CD4 T cell count (cells/µL) (%)**								
**< 200**	4(50.0)	62(63.3)	218(72.7)	286(68.8)	322(71.9)	218(72.9)	136(70.1)	1246(70.7)
**≥200**	4(50.0)	36(36.7)	82(27.3)	130(31.2)	126(28.1)	81(27.1)	58(29.9)	517(29.3)
**ART (%)**								
**2 NRTIs +1 IP**	3(37.5)	11(11.2)	37(12.3)	61(14.7)	61(13.6)	48(16.0)	30(15.5)	251(14.2)
**2NRTIs +1 NNRTI**	5(62.5)	87(88.8)	263(87.7)	355(85.3)	387(86.4)	251(84.0)	164(84.5)	1512(85.8)

**ART**: antiretroviral therapy; **NRTIs**: nucleoside reverse transcriptase inhibitors; **NNRTI**: non-nucleoside reverse transcriptase inhibitor; **PI**: protease inhibitor.

***Biological and therapeutic characteristics:*** HIV-1 infection and combined HIV-1+2 infection respectively represented 94.2% and 4.0% of cases. At the beginning of the antiretroviral combined therapy, the median of CD4 cell count was 126 c/µl (IQR: 70-213 cells/µl); with extremes (1- 412 cells/µl); more than two-thirds (70.7%; n=1,246) of patients had less than 200 CD4 cells count. The combination of two nucleoside reverse transcriptase inhibitors (NRTI) plus one non-nucleoside inhibitor of reverse transcriptase (NNRTI) was prescribed as first line therapy in 85.7% of cases and 2 NRTI+1 PI (protease inhibitor) in 14.2% of cases. Nevirapine was the most commonly prescribed (54.1%) medication. Out of the 1,763 women of childbearing age, 558 (31.6%) were under ART which includes Efavirenz. The average duration of the follow up period was 29 months (IQR: 13-48 months) with extremes (1 - 74 months).

***Incidence of pregnancies in the cohort:*** Throughout the study, 222 pregnancies were observed among 1,763 women, with a follow-up of 4,639 women-years, corresponding to an incidence density of 5 pregnancies for 100 women-years (IC 95%: 4, 2-5, 5). The median time between the beginning of the ART and the occurrence of the pregnancy was 19 months (IQR: 9-36 months). The incidence of the pregnancy increased with the length of the exposure to the treatment; it was respectively 1.4%, 4.2% and 9.1% women-years at 6, 12 and 24 months of treatment. Among the 558 patients under Efavirenz, 46 (8.2%) became pregnant. Only 18 had their treatment changed due to the pregnancy. Efavirenz was replaced by Nevirapine or Lopinavir boosted with Ritonavir.

***Outcome of pregnancies:*** No information on the outcome 8 out of 222 pregnancies (3.6%) was recorded. The outcome of 214 pregnancies was registered and resulted in 9 cases of abortion (4.0%); with a median gestational age of 12 weeks of amenorrhea (IQR, 8-13); 205 pregnancies (92.4%) resulted in childbirths including 15 premature childbirths. Natural delivery represent 90.2%, (185/205) compared to 9.8% (20/205) caesarean sections. The frequency of live births and stillborns was respectively 94.0% (193/205) and 6.0% (12/205). Babies’ median weight was 2,800g (IQR: 2,550g; 3,100g); 30 babies (representing 14.6% of live births) were born with less than the median weight (below 2,500g).

***Fertility rate of HIV infected patients:*** Table 2 provides details of the age-standardized fertility rate and standardized incidence ratios. The standardized crude fertility rate in our cohort was 45 live births for 1,000 women-years. The fertility rate has changed in all age groups of PLWHA compared to the baseline, except for the 15 to 19 years old group. The standardized incidence ratios (SIR) are low and the overall decrease in fertility was 66.0% compared to the entire population. According to the age groups, this decrease in fertility varies from 15.0% to 70.0%.

**Table 2:** Number of newborns, incidence ratio and age-standardized fertility rate of women on ART at the Bobo-Dioulasso Day Care Unit, Souro Sanou Teaching Hospital, Burkina Faso, 2005-2011*

	Baseline population	People living with HIV			
Age (years)	Fertility rates for 1000 women-year	Number of expected live newborns	Number of newborns observed	Standardized Incidence Ratio	Standardized fertility rates for 1000 women-years
15 - 19	69	1	1	1.00	69.0
20 - 24	167	40	18	0.45	75.1
25 - 29	186	135	43	0.32	59.5
30 - 34	180	198	60	0.30	54
35 - 39	120	144	47	0.33	39.6
40 - 44	48	39	18	0.46	22.1
45- 49	15	8	6	0.75	11.2
Total	132	565	193	0.34	45.0

* Indirect standardization using Demographic and Health Survey, Burkina Faso, 2010

## Discussion

This retrospective study has some limitations. Despite it is condemned by the law in Burkina Faso, pregnancy termination is still performed. Therefore, we cannot assert that all cases of pregnancy have been registered. However, this innovative study made it possible to assess the frequency of pregnancies among patients on ART in Burkina Faso. The incidence of pregnancy was 5 for 100 women-years. However, this number is below most of rates found in African studies [8, 9, 20–23] with a frequency ranging from 7.9 to 24.6 for 100 women-years. The incidence of pregnancy could vary from one study to another depending on how patients are selected and on the pregnancy diagnosis criteria as well. The relative high number of our patients (median age 35) and the low proportion of people living as husband and wife (44.6%) could account for the low frequency of pregnancies in our study. Performing systematic and regular pregnancy tests would help determine the number of pregnancies ending up into miscarriage. The proportion of abortion was 4.0% (n=9) in our study. It is lower compared to that reported by Homsy et al. [24] in Uganda and Bussman et al. [8] in Botswana, respectively with 58.6% and 42.3%. High abortion prevalence in the two later surveys was due to the high number of induced abortion. The information collected as part of our study does not enable us to differentiate spontaneous abortion (miscarriages) from induced abortions. In our survey, live births represent 94.0%. A total of 12 stillborns were recorded, representing 6.0% of pregnancies. Our results are close to those found by Ekouevi et al. [25] in Côte d'Ivoire and Liu et al [26] in South Africa and Zambia. The median weight of babies born from mothers under ART was 2,800g, whereas the lower weight was 14.6% for live babies. The finding of our investigations is close to those made by researchers from sub-Saharan Africa [8, 25–27]. Some of them related low weight at birth to mothers undergoing an antiretroviral treatment [28]. However, the findings of studies conducted on this issue remain quite controversial. This study shows a 15 to 70% decrease in fertility rates among HIV infected women compared to the overall population, except for the age group of 15 to 19. This corroborates the findings from existing literature papers [10, 29]. Poor information provided to patients in relation with the impact of antiretroviral treatment on reduction of mother-to-child transmission and high frequency of non severe amenorrhea during the infection could account for the decrease in fertility among those women. Low fertility can also be explained by the increased use of condoms by HIV infected women fearing to pass on the infection [30]. PLWHA care services should provide them reproductive health care in order to help those women exercise their sexual and reproductive rights in the best and safe conditions both for the couple and the baby to be born [31, 32].

## Conclusion

This study highlights a low pregnancy incidence among women under art compared to the population in general. Women who became pregnant while on ART treatment delivered live babies in most cases. The care package for HIV infected women of childbearing age should include reproductive services. Women willing to give birth must be supported through counseling and with health care to ensure better conditions for the couple and the fetus. The follow up of such pregnancies should involve specialists of infection, obstetricians, and pediatricians collaborating for more favorable outcome.

### What is known about this topic

- Before the era of high active antiretroviral therapy (HAART), HIV infection evolved inescapable way to short or medium term towards death.
- Today, thanks to HAART mother to child transmission of HIV is practically zero and PLWHA in therapeutic success have a quality and a life expectancy comparable to persons HIV negative.
- However reproductive issues are little discussed by practitioners working in HIV care centers in Burkina Faso. The latter being shared between the obligation to promote the prevention of horizontal transmission of HIV (wearing a condom) and the need to inform patients about the possibility of conceiving an HIV-negative child with ARV treatment.

### What this study adds

- This study highlights a low pregnancy incidence among women under ART compared to the population in general.
- The standardized incidence ratios (SIR) are low and the overall decrease in fertility was 66.0% compared to the entire population.
- Women who became pregnant while under ART delivered live babies in most cases.
